# Electrolysis Extraordinary

**Published:** 1874-08

**Authors:** 


Electrolysis Extraordinary!—A gentleman who had been suffering from a superabundance of adipose tissue, consulted the physician, asking for relief from its burden. The doctor told him he could relieve him if he would consent to a painful operation. The gentleman consented, and with the medical practitioner entered the telegraph office at that place. The fat man was requested to remove his coat and vest, after which the physician surrounded him with wires, attaching the ends to a powerful battery. At a signal from the doctor, Manager Eddy let on the current. The patient writhed and twisted when he felt the current passing around him; still, he stood like a martyr. Presently he began to shrink; he grew smaller and smaller; his clothing hung in bags about his fast diminishing form; the doctor felt much pleased at the result of his experiment; while the formerly fat man's joy was very great, although he seemed to be suffering acute pain. All of a sudden there was heard a loud clicking at the instrument, as if Pandemonium's great hall had been let loose. The operator sprang quickly to answer the call. He ascertained it was from the New York office. He quickly asked: "What's up?" An answer came back as if some infuriated demon was at the other end of the wire: "What in thunder are you about? Cut off your wires quick—you are filling the New York office with soap grease!"—*Criterion.*

